# Supplementary material for: Characterization of Interactions between CTX-M-15 and Clavulanic Acid, Desfuroylceftiofur, Ceftiofur, Ampicillin, and Nitrocefin
Source: Int J Mol Sci. 2022 May 7;23(9):5229. doi: 10.3390/ijms23095229 (PMC9100253; doi:10.3390/ijms23095229)

Supplemental data:

Figure S1. Nitrocefin binding pocket of CTX-M-15. Hydrogen bonds are depicted by black dotted lines and nitrocefin is in red.

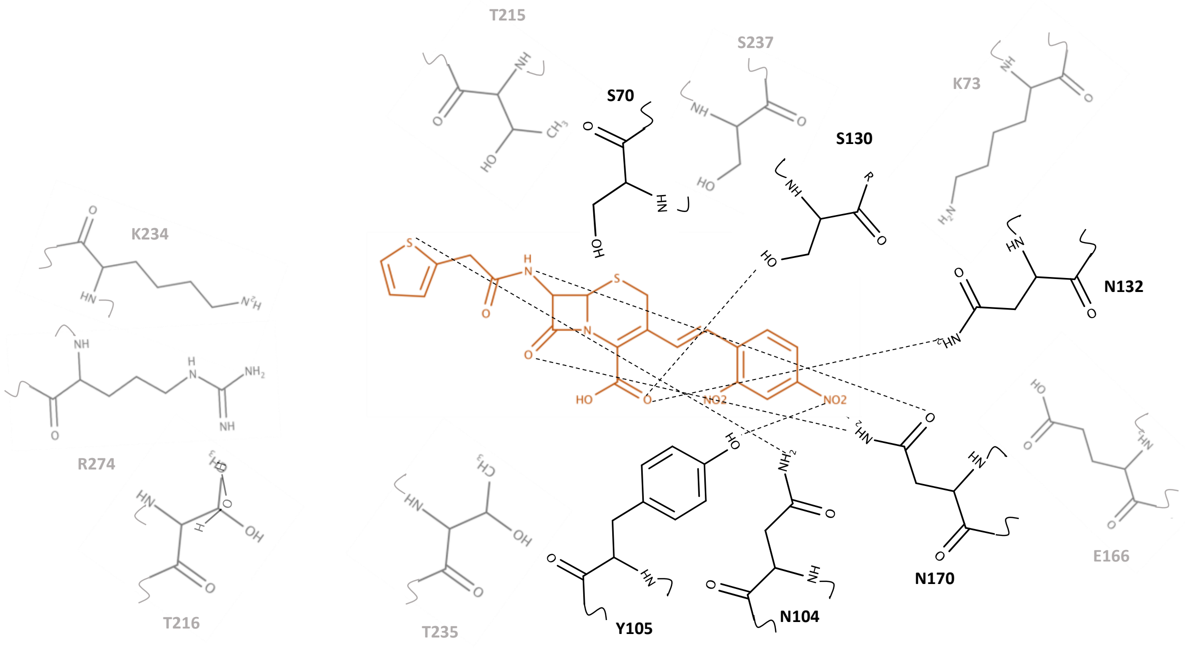

Figure S2. Ceftiofur binding pocket of CTX-M-15. Hydrogen bonds are depicted by black dotted lines and ceftiofur is in red.

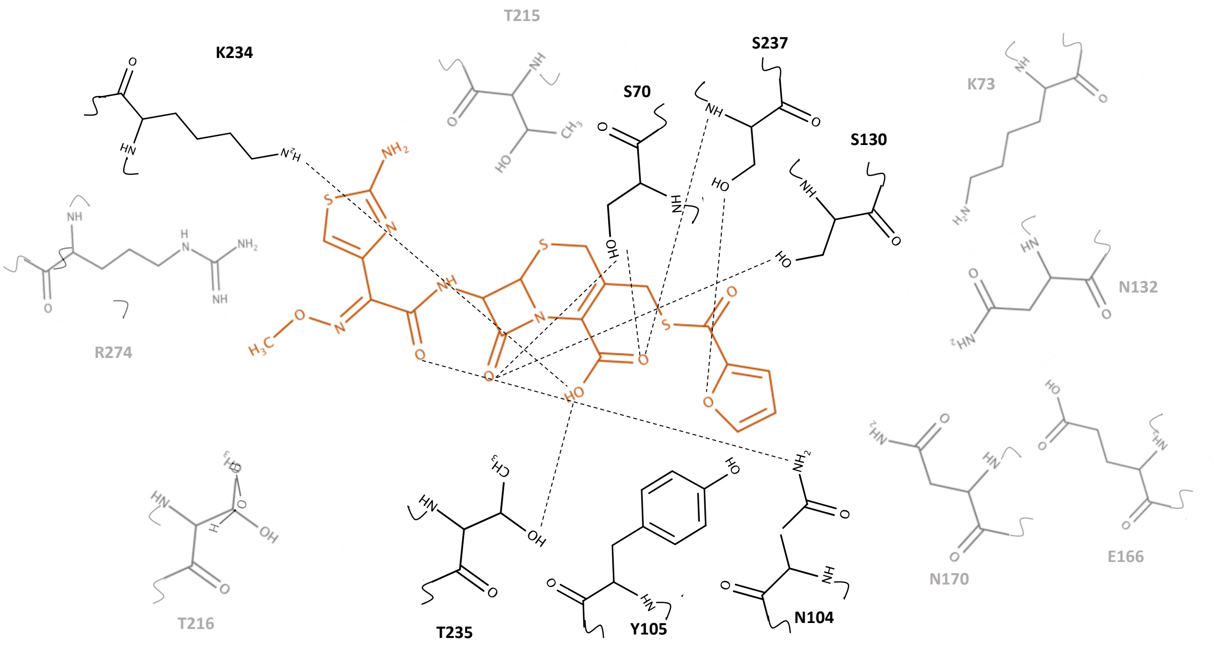

Figure S3. The hydrolysis mechanism for ampicillin by CTX-M-15. The arrows displayed the electron and proton transferring. MarvinSketch v.17.28. was applied to generate these figures. **I)** A shared proton is transferred between Glu166 and Lys73, and ampicillin is in the binding pocket. **II)** The proton is transferred to Glu166 and a neutral Lys73 abstracted a proton from Ser70. The activated Ser70 attacks the carbonyl carbon of the β-lactam ring. **III)** The acyl-enzyme intermediate is generated by a covalent bond between the sidechain hydroxyl of Ser70 and the carbonyl carbon of β-lactam ring. The bridgehead nitrogen abstracts a proton from Ser130 and the shared proton is transferred from Glu166 to Lys73, and finally to Ser130. **IV)** A catalytic water is activated by the negatively charged Glu166 and the activated water attacks to carbonyl carbon in the acyl-enzyme intermediate. **V)** The tetrahedral transition state is broken in the diacylation reaction via the protonation of Ser70 by Lys73. The Lys73 abstracts the proton from Glu166. **VI)** The hydrolyzed ampicillin is released from binding pocket and enzyme activated again.

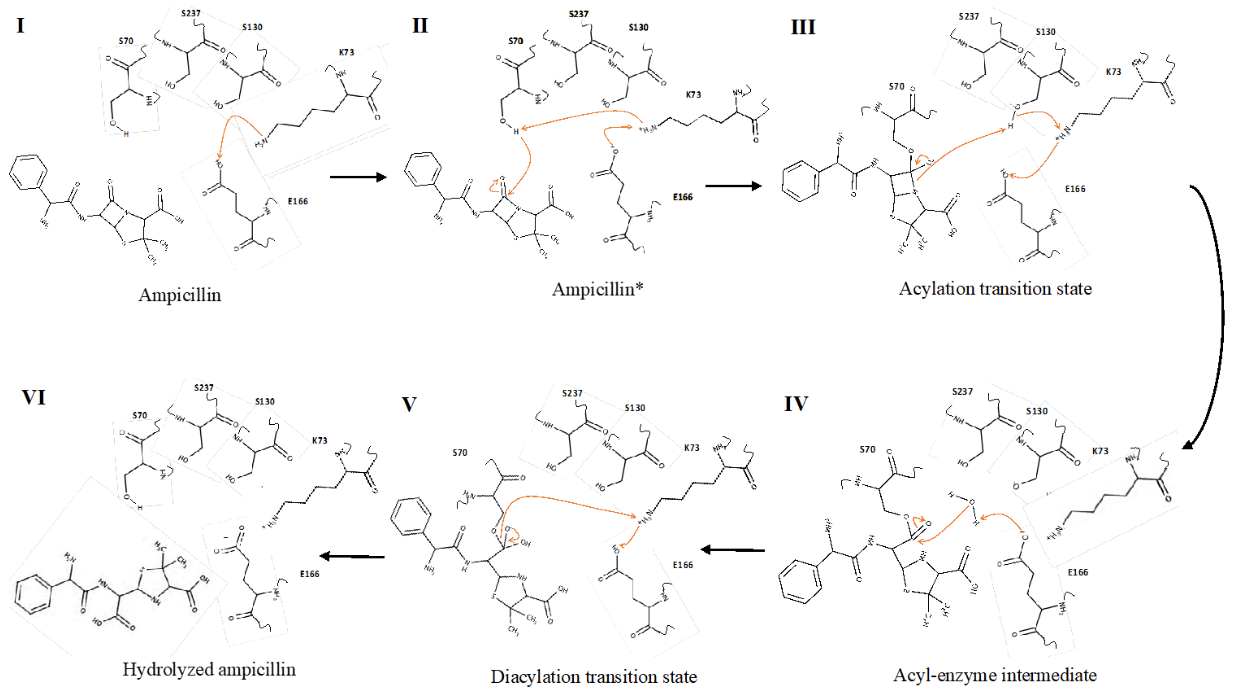

Figure S4. The enzyme kinetic graphs of WT-CTX-M-15 and S70A-CTX-M-15. Kinetic assay for dependence on (a) DFC, (b) ampicillin, (c) ceftiofur, (d) nitrocefin with a constant concentration of enzyme. Figures were generated using GraphPad Prism v. 9.3.1 (GraphPad Software).

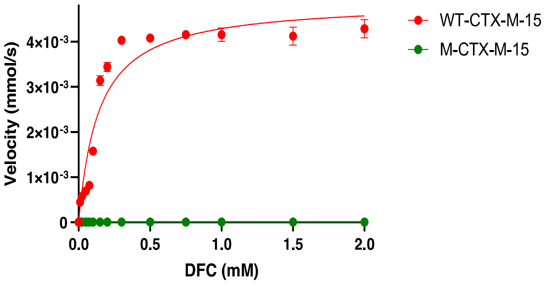

(a)

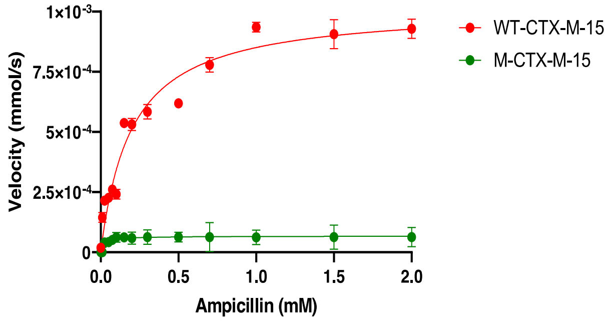

(b)

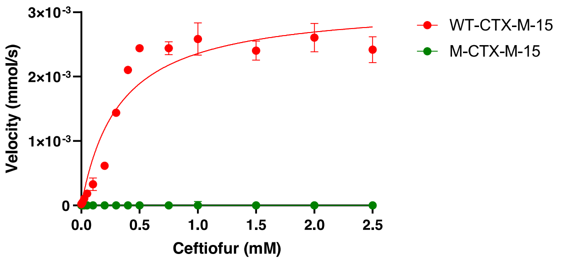

(c)

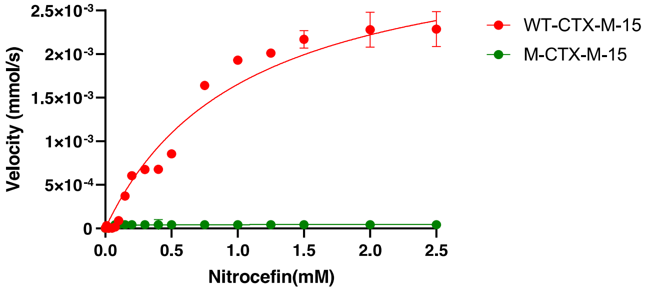

(d)

Figure S5. Lineweaver-Burk plot of CTX-M-15 displaying a mixed inhibition of clavulanic acid versus ceftiofur (0-1.5 mM). Data points are mean values taken in triplicate showing standard deviations.

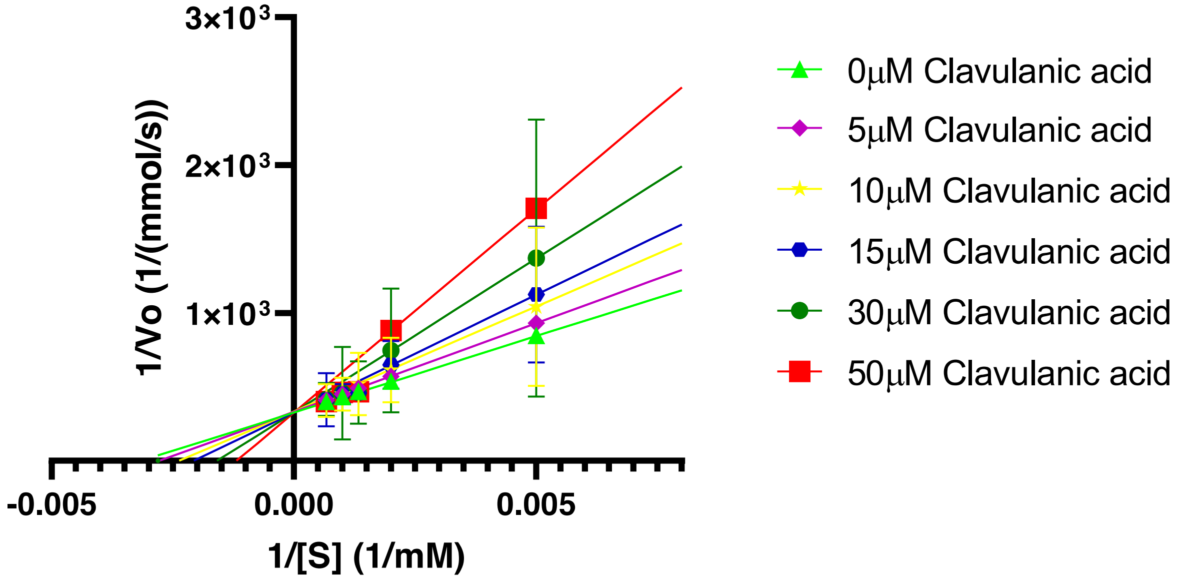

Figure S6. The graphs of isothermal titration calorimetry of S70A-CTX-M-15 with four β-lactams and one β-lactamase inhibitor. (a) DFC; (b) ampicillin; (c) ceftiofur; (d) nitrocefin; and (e) clavulanic acid. The graphs were generated with originlab/origin7.

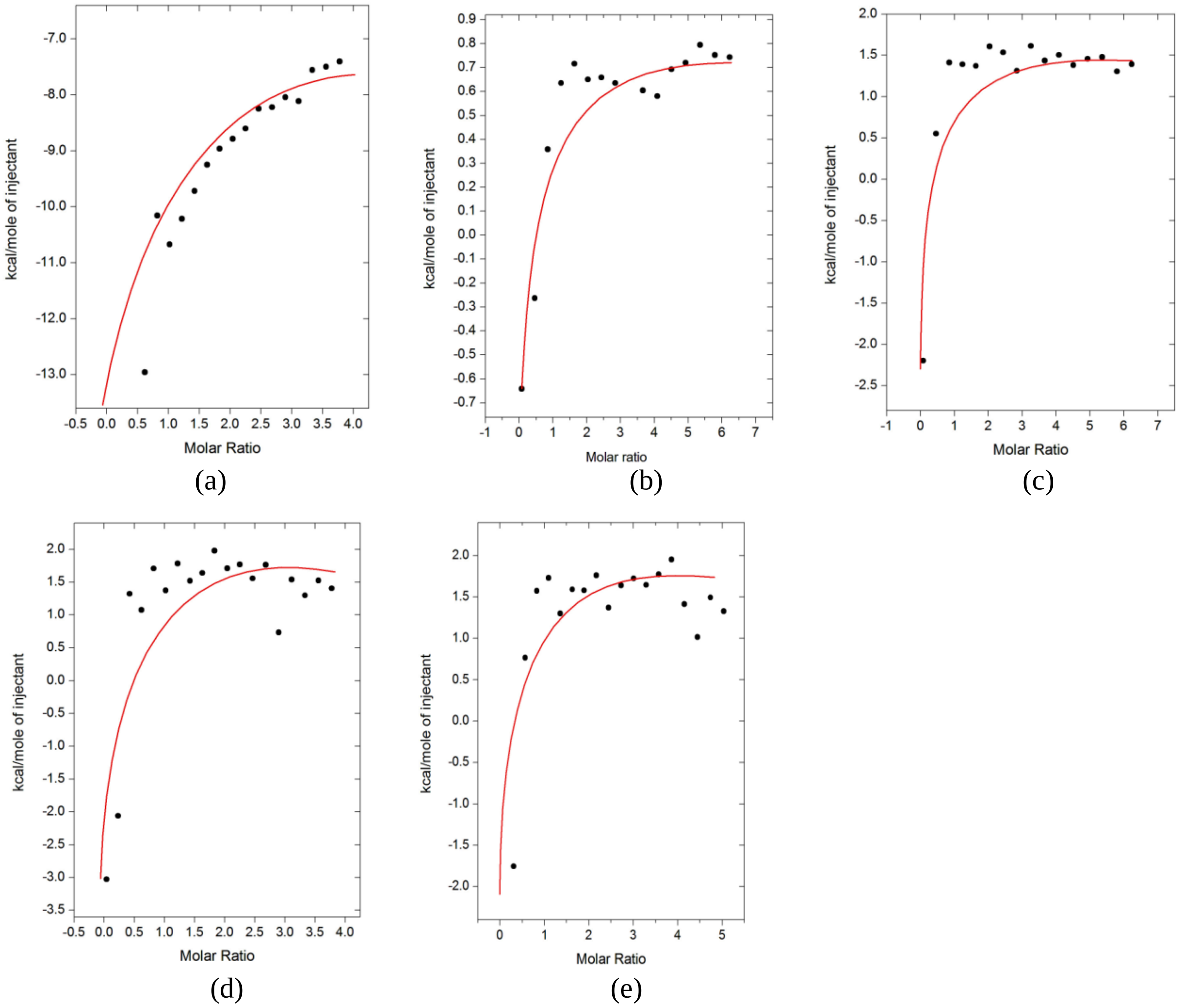

Figure S7. A view of the electron density around the clavulanic acid at the $2F_o$-F_c map contoured at 1.0, 2.0, and 3.0 σ (a) the map contoured at 1.0 σ; (b) the map contoured at 2.0 σ; (c) the map contoured at 3.0 σ (electron density appeared in some atoms of the ligand and amino acids). Bound clavulanic acid is a greenish, and amino acids are in yellow and pink. Figures were made with Coot 0.8.9.3-pre EL (revision count 8011).

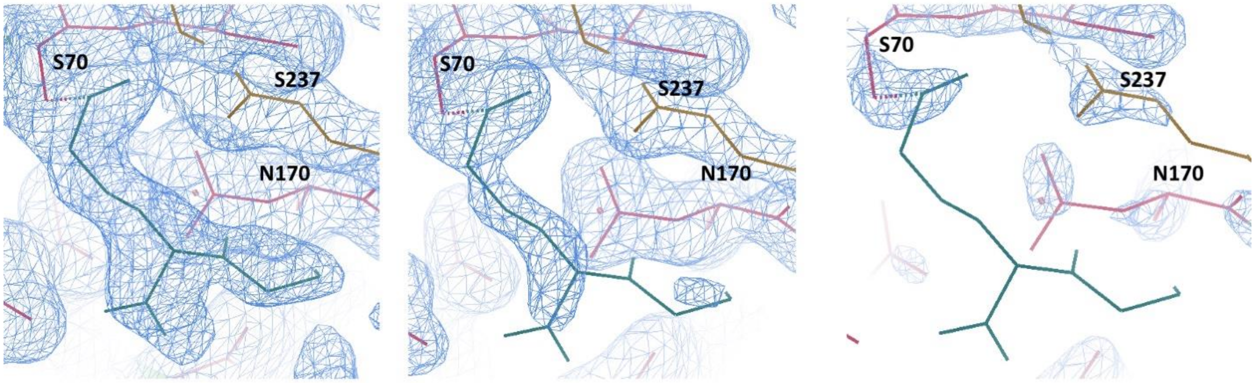

(a) (b) (c)

Figure S8. Ligand-binding pocket of CTX-M-15. Superposition of apo CTX-M-15 (beige) and clavulanic acid-CTX-M-15 (pastel blue). The covalently bound clavulanic acid is yellow. The UCSF Chimera v1.13.1. built this figure.

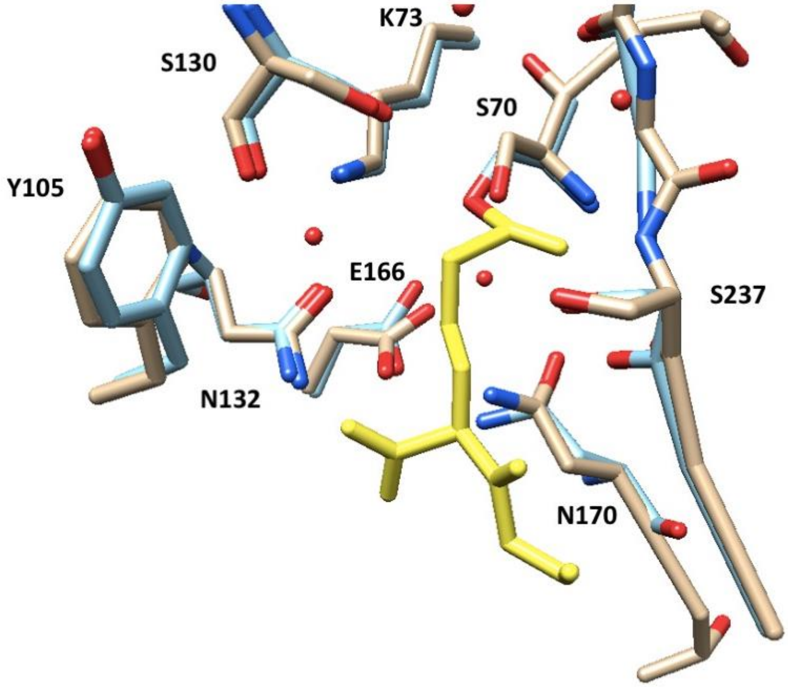

Figure S9. A view of the $2F_o$-F_c map around the bound ampicillin contoured at 1.0, 2.0, and 3.0 σ. (a) the map contoured at 1.0 σ; (b) the map contoured at 2.0 σ; (c) the map contoured at 3.0 σ (electron density appeared in some atoms of the ligand and amino acids). The bound ampicillin is dark blue, and amino acids are in yellow and pink. Figures were made with Coot 0.8.9.3-pre EL (revision count 8011).

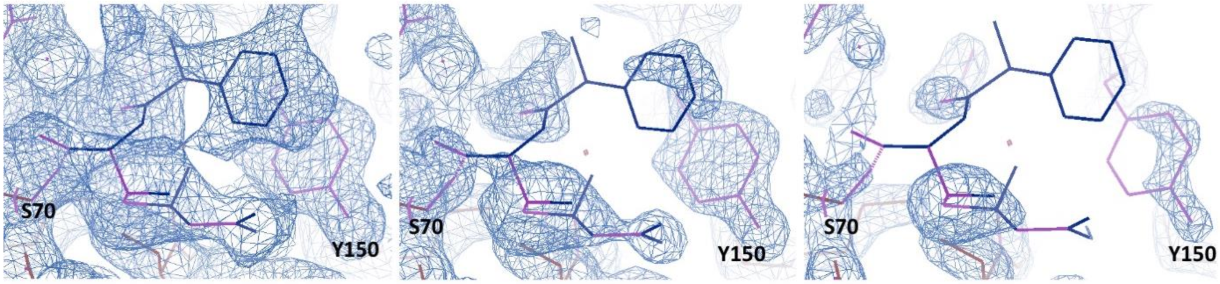

(a) (b) (c)

Figure S10. Ligand-binding pocket of CTX-M-15. Superposition of apo CTX-M-15 (beige) and ampicillin-CTX-M-15 (pastel blue). The covalently bound ampicillin is yellow. The UCSF Chimera v1.13.1. built this figure.

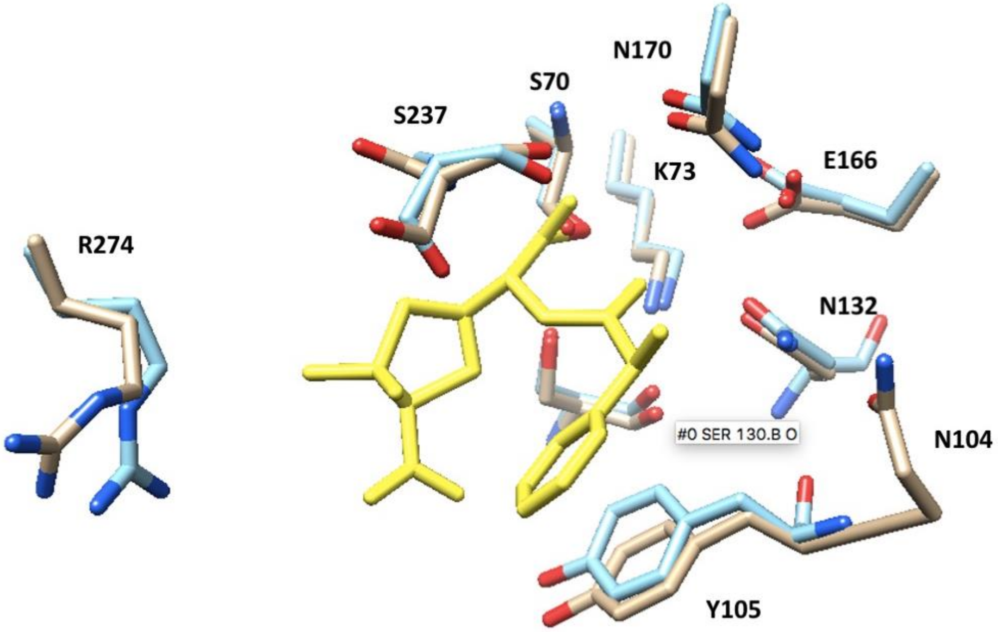

Figure S11. A view of the $2F_o$-F_c map around the bound DFC contoured at 1.0, 2.0, and 3.0 σ; (a) the map contoured at 1.0 σ; (b) the map contoured at 2.0 σ; (c) the map contoured at 3.0 σ(electron density appeared in some atoms of the ligand and amino acids). The bound DFC is maroon, and amino acids are in gold and dark purple. Figures were made with Coot 0.8.9.3-pre EL (revision count 8011).

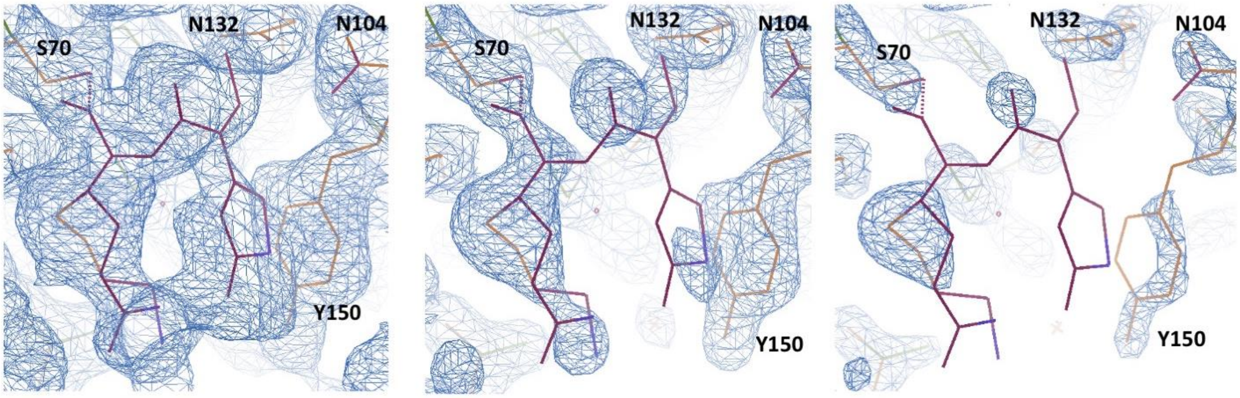

(a) (b) (c)

Figure S12. Ligand-binding pocket of CTX-M-15. Superposition of apo CTX-M-15 (beige) and DFC-CTX-M-15 (pastel blue). The covalently bound DFC is yellow. The UCSF Chimera v1.13.1. built this figure.

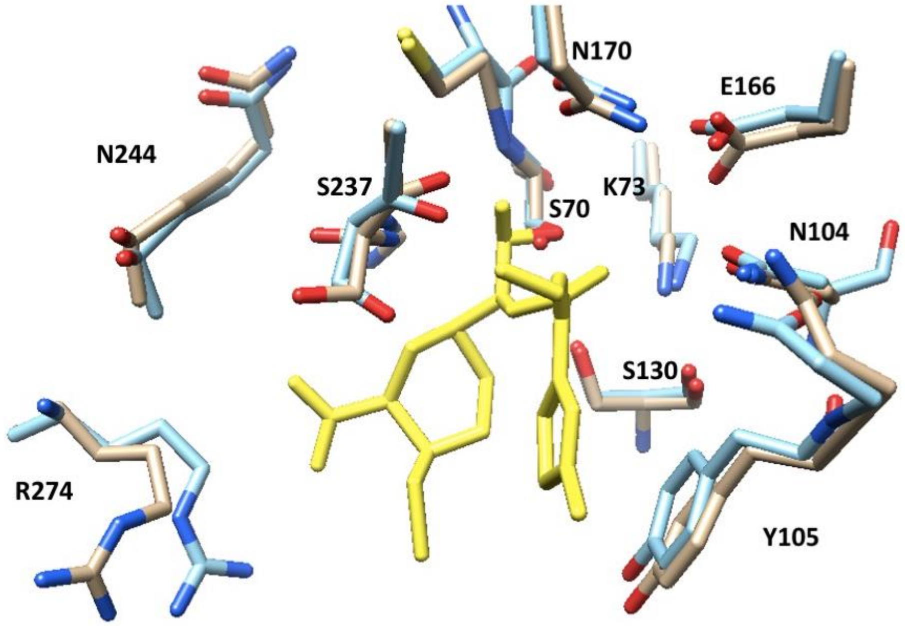

Figure S13. The hydrolysis mechanism of DFC by CTX-M-15**.** The electron and proton transfers are indicated by arrows. **I)** DFC binding and proton transferring from Glu166 to Lys73. **II)** Glu166 abstracts a proton from Lys73, a neutral Lys73 activates Ser70 by deprotonating its sidechain hydroxyl. The nucleophilic Ser70 attacks the carbonyl carbon of b-lactam ring. **III)** The Ser70 is covalently bonded to DFC making an acylation transition state. The bridgehead nitrogen abstracts a proton from Ser130 and the shared proton is transferred from Glu166 to Lys73, and finally to Ser130. **IV)** A negatively charged Glu166 abstracts a proton from a water molecule and activates it to hydrolyze the acyloxyl linkage in the acyl-enzyme intermediate. **V)** Ser70 is protonated by Lys73 through the breakdown of tetrahedral diacylation transition state. The Lys73 abstracts the proton from Glu166. **VI)** The hydrolyzed DFC is then released from binding pocket and enzyme is activated again.

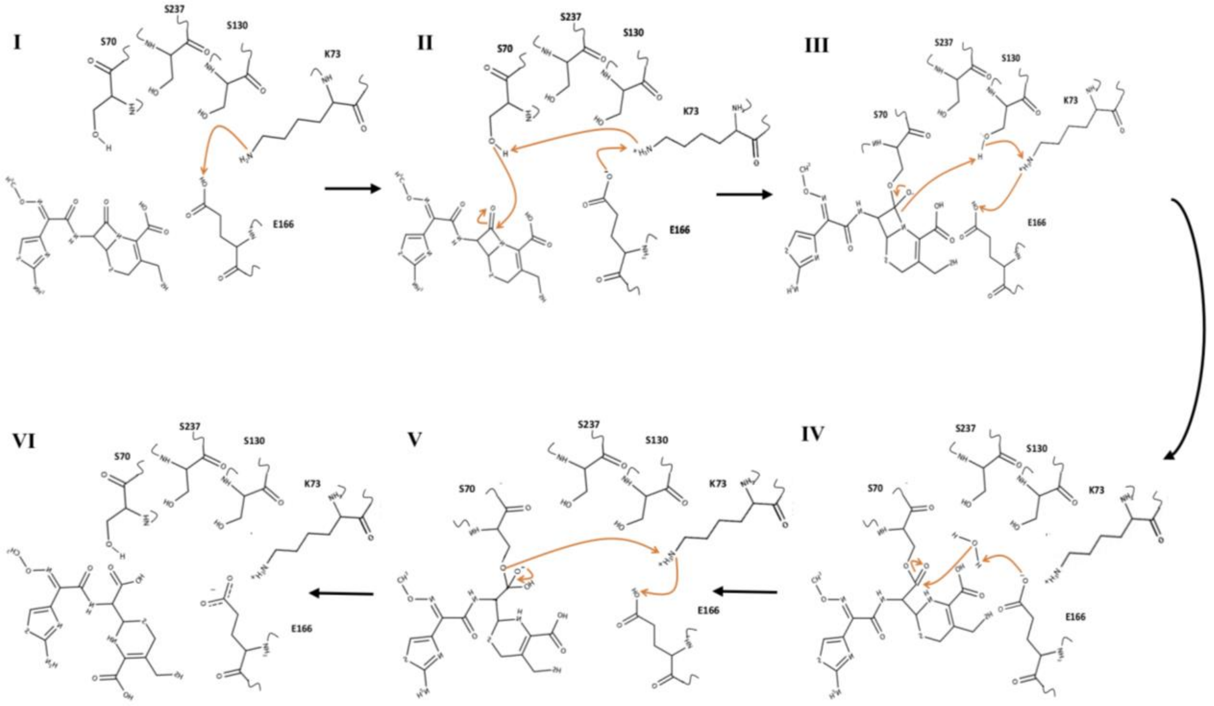

Supplement: Supplementary file 1 [file ijms-23-05229-s001.zip › ijms-1652688-supplementary.pdf]
